# Simvastatin Efficacy on Endodontic Biofilms: An In Vitro Study

**DOI:** 10.3390/ma17225441

**Published:** 2024-11-07

**Authors:** Carmen María Ferrer-Luque, Marcos Hernández, Carmen Solana, Matilde Ruiz-Linares

**Affiliations:** 1 Department of Stomatology, School of Dentistry, University of Granada, 18071 Granada, Spain; marcoshl16@correo.ugr.es (M.H.); csolana@ugr.es (C.S.); 2 Instituto de Investigación Biosanitaria ibs.GRANADA, 18012 Granada, Spain

**Keywords:** antimicrobial, biofilm, diclofenac, simvastatin, residual activity

## Abstract

The outcome of endodontic therapy is directly related to the control of infection. The aim of the study was to evaluate in vitro the efficacy of Simvastatin (SIM) and diclofenac (DC) solutions on endodontic biofilms. Methods: Mature biofilms were grown on dentin specimens and put in contact with the solutions to determine their antibiofilm activity. To assess residual antimicrobial activity, the dentin samples were immersed for 5 min in the solutions before microbial infection for 3 weeks. The study groups were: (1) 8% SIM, (2) 4% SIM, (3) 4% DC, (4) 2.5% sodium hypochlorite (NaOCl), and (5) 0.9% saline solution (SS). Cell viability was evaluated by means of the adenosine triphosphate (ATP) assay and flow cytometry (FC). The data collected were analyzed with the ANOVA test using Welch’s correction followed by the Games–Howell test. The level of statistical significance was *p* < 0.05, and statistical analysis was performed using SPSS 23.0. Results: All study groups reduced the biofilms significantly with respect to the control. The highest reduction percentage was obtained by 2.5% NaOCl, followed by SIM 8% and 4%, without statistically significant differences. In terms of residual activity, the 4% DC solution obtained a higher percentage of dead cells. Conclusion: Solutions of 4% and 8% SIM, and 4% Diclofenac, show antimicrobial and residual activity against multispecies endodontic biofilms.

## 1. Introduction

The success of endodontic treatment is fundamentally related to the reduction and elimination of most microorganisms present in the complex root canal system [1].

In pulp infections where there is no periapical involvement, the main requirement to achieve healing is asepsis during treatment, regardless of the clinical protocol used [2,3]. Yet in cases of apical periodontitis, the presence of bacteria organized as microbial biofilms adhered to the dentin of the root canal, and damage to the adjacent periradicular tissues [4,5], determine the need for infection control over time, to resolve the pathological process and restore the health of the affected tissues [6,7].

Antimicrobial solutions—mainly sodium hypochlorite and chlorhexidine, and chelating agents [8,9,10,11]—are used to achieve this purpose [12]. Also, intracanal medications, including calcium hydroxide pastes [13], antibiotics and nonsteroidal anti-inflammatory drugs, either in solution [14,15] or gel form [16], could contribute to disinfection during endodontic treatment. Furthermore, an effective final irrigation protocol is an important step in order to ensure residual antimicrobial activity, i.e., to maintain the achieved disinfection effects after root canal preparation [8,9].

Substances such as statins, drugs used to control cholesterol levels in the body, are being investigated for their anti-inflammatory and immunomodulatory properties [17]. Their efficacy as anti-infective agents, indicated more than a decade ago [18], has been demonstrated against a variety of standard bacterial strains and clinically isolated bacteria [17,18,19,20]. The mechanism of antimicrobial action of statins appears to be related to the reduction in cell growth [19], with decreased biofilm growth and extracellular polysaccharide production [17].

Among the different statins tested, atorvastatin and simvastatin (SIM) show noteworthy antibacterial potential [19,20]. Topical application of SIM appears effective in preventing or treating wound infection caused by *Staphylococcus aureus* [21]. In dentistry, it has demonstrated its effectiveness against *Porphyromonas gingivalis*, efficiently inhibiting the growth and development of established biofilms, making it a possible adjuvant agent in the treatment of chronic periodontitis [22]. In addition, SIM is a statin that may promote alveolar bone regeneration [23]. Local administration of SIM in bone defects in rats led to improved bone formation between the second and fourth week of the healing period [24]. Still, the impact on pre-osteoblast cell growth and osteogenic differentiation of DC is debatable [25,26].

It is known that NaOCl is the main irrigation agent during root canal preparation, but because it lacks substantivity [27], other agents should be tested to determine their usefulness as final irrigating solutions. The objective of the present study is to evaluate and compare in vitro the antimicrobial capacity and the residual activity of three solutions against endodontic biofilms: 4% and 8% SIM, and 4% DC. The null hypothesis was that SIM and DC solutions tested have not the same antimicrobial and residual activity

## 2. Materials and Methods

The protocol of this in vitro study was approved by the Ethics Committee of the University of Granada, Spain (N° 1076 CEIH/2020); informed consent was obtained from all patients before collection of the microbiological samples or extracted teeth. The sample size was estimated based on studies comparing the efficacy of antimicrobial agents against microbial biofilms grown in dentin [11,15,16,27]. In this study, that sample size was estimated to be n = 20/per group: 10 specimens for antimicrobial activity and 10 specimens for residual activity.

### 2.1. Antibiofilm Efficacy

Polymicrobial biofilm formation was performed on sterile dentin samples. To this end, 30 newly extracted, non-carious, single-rooted human teeth were selected and stored in thymol at 4 °C until use. The crowns of the teeth were sectioned at the cementoenamel junction, and the middle and apical thirds of the root were discarded.

The coronal third of the root was divided longitudinally into two halves, and the cementum of each half, as well as the inner part of the root, was polished with 500- to 2000-grit silicon carbide discs to create a flat surface. The size was adjusted using a caliper to obtain 60 dentin volumetric units of 4 × 4 × 1 mm (width × length × height). The smear layer formed during sample preparation was removed with 17% ethylenediamine tetraacetic acid (EDTA) for 3 min. Subsequently, the samples were washed with distilled water for 10 min and sterilized in an autoclave for 20 min at 121 °C. The sterility of the dentin samples was checked by incubating them in 5 mL of trypticase soy broth (TSB) (Scharlau Chemie SA, Barcelona, Spain) at 37 °C for 24 h to verify the absence of turbidity.

Microbial samples for biofilm formation were collected from the root canals of necrotic teeth. Microbial samples were mixed in TSB and incubated anaerobically at 37 °C for 24 h. Cell density was adjusted in a spectrophotometer to a concentration of approximately 3.0 × 10^7^ colony-forming units per milliliter (CFUs/mL). Infection of dentin samples was performed in 24-well plates, inoculated with 200 μL of the microbial suspension and 1.8 mL of sterile TSB. Sterile dentin blocks were dipped into the inoculated wells and incubated for 3 weeks at 37 °C in an anaerobic atmosphere. The medium was renewed once a week.

After the biofilm formation, the dentin samples were washed with saline solution and randomly assigned to receive the antimicrobial treatment. The study groups (n = 10) were as follows: group (1) 8% SIM; group (2) 4% SIM; group (3) 4% DC; group (4) NaOCl 2.5%; and group (5) 0.9% SS (control).

The dentin blocks were immersed in 120 μL of the antimicrobial solutions for 5 min. Then, the samples were placed in Eppendorf tubes with 200 μL of TSB, vortexed for 10 s and sonicated for 10 min to ensure the recovery of the biofilms. For the control group, a similar procedure was followed, except that there was no exposure to any antimicrobial. The microbial viability was evaluated by means of the adenosine triphosphate (ATP) assay and flow cytometry (FC).

The ATP levels contained in the suspension of recovered biofilms were determined with the BacTiter-Glo Microbial Cell Viability Assay (BacTiter-Glo; Promega, Madison, WI, USA), according to the manufacturer’s instructions. The reagent is based on the properties of a thermostable luciferase that uses ATP from viable cells to generate light photons. The formulation of the reagent determines the lysis of bacterial cells and the generation of quantifiable light. The luminescent signal is proportional to the amount of ATP present, and this is directly proportional to the number of viable cells, expressed as Relative Light Units (RLUs). For bacterial count determination, 100 μL of bacterial suspension was added to 100 μL of BacTiter-Glow reagent in a 96-well white plate (Greiner, Monroe, NC, USA), followed by incubation at room temperature for 5 min. The luminescence produced was measured with a luminometer (GloMax; Promega), and results were expressed as absolute value and percentage reduction in RLUs of the specimens with respect to the control using the formula: (1 − [RLUs test/RLUs control]) × 100.

For FC, 100 µL of the microbial suspension was labeled with the cell viability kit LIVE/DEAD (BacLight; Invitrogen, Eugene, OR, USA), as previously reported [16]. The tube was placed in the FACSCanto flow system (FACSCanto II cytometer, Becton, Dickinson, Madrid, Spain), and the results were analyzed with the software of the cytometer (FACSDiva Version 6.1.3., Becton, Dickinson), which made it possible to obtain a graph of two-dimensional points representing the different cell populations within the microbiological sample, with damaged membranes (dead) or undamaged ones (live). Results were expressed as absolute values of damaged cell membranes per mL and dead cell percentage.

Images obtained by confocal laser scanning microscopy (CLSM) served for in situ visualization of the residual biofilm in dentin (n = 2/group).

### 2.2. Residual Antimicrobial Activity

A total of 25 single-rooted teeth were used, from which 50 dentin specimens were obtained, processed as described above. The study groups (n = 10/group) and microbiological suspension used for this experiment were the same as for the determination of antibiofilm activity.

To assess the residual activity of the solutions, all dentin specimens were dried with paper discs and then immersed for 5 min in the wells of a 96-well microtiter plate (Nunclon Delta Surface, Nunc, Roskilde, Denmark) containing 120 µL of the different solutions tested.

After 5 min, the specimens were dried and transferred to a microtiter plate with 200 µL of the microbial suspension. The plate was then incubated for 3 weeks at 37 °C in an anaerobic atmosphere. The residual antimicrobial activity of the solutions was determined by the ATP test and FC described above.

### 2.3. Statistical Analysis

Before the statistical analysis, the data of percentage reduction were subjected to the Anscombe transformation. The RLU values followed a normal distribution in all study groups according to the Shapiro–Wilk test, but there was no homogeneity of variances with the Levene test. Comparisons were made by means of the ANOVA test with Welch correction followed by the Games–Howell test. The level of statistical significance was set at *p* < 0.05; statistical analysis was performed using SPSS 23.0 (SPSS Inc., Chicago, IL, USA).

## 3. Results

**Figure 1:**
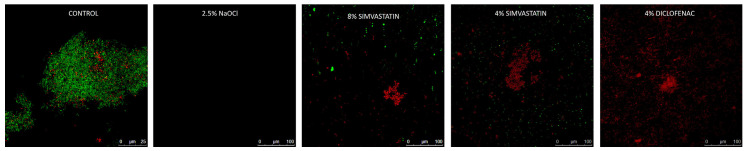
Representative confocal laser scanning microscopy images of 3-week polymicrobial endodontic biofilms treated with the experimental solutions.

The bioluminescence results of the antimicrobial activity of the solutions are shown in Table 1. All study groups showed a statistically significant decrease in RLUs compared to the control. The highest percentage of microbial reduction was obtained by the 2.5% NaOCl group, followed by the 8% and 4% SIM groups, without significant differences between the two concentrations, but the difference was significant when compared to a 4% DC solution. Similar findings were obtained by the FC, in agreement with CSLM representative images of residual biofilms (Figure 1).

The results of the residual antimicrobial activity are shown in Table 2. Over a 3-week period, 4% DC showed the lowest percentage of microbial viability, followed by 4% and 8% SIM. The lowest percentage of death was seen for the 2.5% NaOCl group, not statistically different from the control group.

## 4. Discussion

The outcome of endodontic therapy is directly related to the control of infection. Root canal treatment is the procedure used to prevent apical periodontitis or, once established, to treat and resolve it. Although chemical–mechanical debridement is the main strategy for resolving the infectious process [8,11], topical administration of medications can prove an effective adjuvant therapy [13]. Given the antibiotic resistance and persistent nature of microbiota after root canal treatment, the use of alternative substances for disinfection may involve repositioning drugs that already exhibit satisfactory results [17,22].

The present study evaluated the antimicrobial efficacy of 4% and 8% SIM solutions, and 4% DC, against mature polymicrobial biofilms, formed during three weeks in root dentin, allowing for a closer approximation to clinical reality. Cell viability was evaluated by bioluminescence, determined by the detection of adenosine triphosphate (ATP) present in microbial cells. This rapid method makes it possible to estimate the presence and quantity of viable bacteria (not necessarily capable of growing in a medium) during root canal treatment [28].

The null hypothesis was partially rejected since a 4% and 8% SIM solution have similar antimicrobial and residual activity, but different from a 4% DC solution. The results obtained demonstrate the effectiveness of all tested solutions against polymicrobial biofilms, giving statistically significant differences with respect to the control (SS 0.9%). The highest percentage of viable cell reduction, as expected, was obtained by the 2.5% NaOCl solution (99.33%), followed by both 8% and 4% SIM solutions (respectively, 87.10% and 86.21%) and DC (76.34%). Previously reported is a reduction percentage greater than 93% when 2.5% NaOCl solution was used for 3 min on a mature biofilm of *Enterococcus faecalis* [11]. In our study, the exposure time of 5 min could be responsible for the greater antimicrobial efficacy of the solution.

SIM has demonstrated a broad-spectrum antibacterial activity against important pathogens, both Gram-positive and Gram-negative, once the barrier imposed by the outer membrane of the latter was permeabilized [21,22]. Proteomics and macromolecular synthesis analyses reveal that SIM inhibits multiple biosynthetic pathways and cellular processes in bacteria; in addition to the selective interference of bacterial protein synthesis, it affords anti-inflammatory, immunomodulatory, and antioxidant effects [29]. Its antimicrobial capacity—attributed to the structural alteration of teichoic acid and/or a reduction in the number of alanine residues on the surfaces of Gram-positive bacterial cells—would lead to a lesser formation of biofilms [17] and hinder the formation of the polysaccharide matrix that provides protection [30].

The results obtained here are in line with these reports of the antimicrobial efficacy of SIM, in our case against endodontic biofilms. The RLU values with both SIM concentrations (4% and 8%) were very similar, underlining a high efficacy in reducing the percentage of microbial cell viability. DC at 4% obtained a lower reduction percentage, but not far from that seen for this solution in a recent publication when used at a 5% concentration on polymicrobial root canal biofilm [31]. DC is known to have greater antimicrobial effects than triple and double antibiotic solutions against a monospecies biofilm [15] as well as a multispecies biofilm when used in hydrogel form, when compared with antibiotics or a calcium hydroxide paste [16].

Regarding residual activity, it should be noted that all tested solutions except 5% NaOCl were able to significantly reduce microorganisms after three weeks of contact with the polymicrobial suspension. The highest activity was obtained by 4% DC (greater than with both SIM solutions). In this sense, the addition of 5% and 10% of DC to a calcium silicate cement enhanced their antimicrobial and antibiofilm efficacy on polymicrobial biofilms, in a concentration- and time-dependent manner [32]. The potential of DC to disinfect dentinal tubules [33] may have contributed to the high substantivity encountered here. It should be stressed that the solvent used for SIM and DC was dimethyl sulfoxide (DMSO), an excipient that promotes drug penetration, allowing better dissolution and gradual absorption of the drug and reducing the level of systemic toxicity [24]. Moreover, DMSO has demonstrated antimicrobial properties that could have contributed to the residual activity found in the tested solutions [29]. An additional advantage presented by SIM and DC solutions—whether used as temporary medication or as final irrigation agents—is their anti-inflammatory efficacy, which could help in postoperative pain reduction [34,35].

One limitation of the present study is that the antibacterial evaluation was performed immediately and over a 3-week period. Different solutions may require diverse time scales to demonstrate their optimal effects. In addition, the short- and long-term effects of these drugs on the mechanical and chemical properties of dentin should be elucidated before suggesting them for clinical application.

The elimination of pathogenic biofilms is a great challenge, since their resistance to antibacterials and antibiotics impedes healing and treatment success. SIM and DC appear to be a recommendable alternative for root canal treatment [36]. Further research is needed to establish the optimal concentration, delivery vehicle, and possible combination with other substances, as new antimicrobial agents, for the control of endodontic infections.

## 5. Conclusions

Solutions of SIM at 4% and 8% were found to show antimicrobial efficacy against endodontic biofilms. A 4% DC solution showed the highest residual activity over time.

## Figures and Tables

**Table 1:** Antimicrobial activity of the solutions against endodontic biofilms determined by bioluminescence (Relative Light Units and % reduction) and flow cytometry (dead cells/mL and % death). Mean (SD). n = 10/group.

Groups	RLUs	% Reduction	DEAD Cells/mL	% Death
8% SIM	11,618.26 (4478.08) ^a^	87.10 (4.97)	16,444.60 (1228.53) ^a^	54.83 (4.10)
4% SIM	12,421.80 (5086.06) ^a^	86.21 (5.64)	14,576.20 (5879.19) ^a^	51.80 (18.98)
4% DC	21,309.93 (5290.33) ^b^	76.34 (5.87)	12,163.00 (7108.73) ^a^	36.52 (18.89)
2.5% NaOCl	594.73 (151.69) ^c^	99.33 (0.16)	22,374.10 (1188.28) ^b^	74.01 (2.86)
0.9% SS	90,094.46 (28,647.32) ^d^	-	2111.20 (630.42) ^c^	7.10 (2.05)

Global comparison between groups determined by ANOVA test with Welch’s correction (*p* < 0.001). The same superscript letter, read vertically, indicates differences that were not statistically significant according to the Games–Howell test. SIM: Simvastatin; DC: Diclofenac; NaOCl: sodium hypochlorite; SS: saline solution.

**Table 2:** Residual antimicrobial activity of the solutions after 3 weeks of contact with the polymicrobial suspension. Determination by bioluminescence (Relative Light Units and % reduction) and flow cytometry (Dead cells/mL and % death). Mean (SD). n = 10/group.

Groups	RLUs	% Reduction	Dead Cells/mL	% Death
8% SIM	2,037,428.40 (629,958.41) ^a^	41.62 (18.04)	18,016.10 (3199.92) ^a^	61.55 (9.23)
4% SIM	1,818,984.60 (732,675.89) ^a^	47.88 (21.00)	21,134.50 (4014.06) ^a^	70.39 (13.32)
4% DC	861,202.40 (53,812.60) ^b^	75.32 (1.54)	25,616.00 (1027.18) ^b^	84.70 (3.80)
2.5% NaOCl	3,373,828.10 (175,804.82) ^c^	3.33 (5.03)	7173.70 (1732.71) ^c^	23.90 (5.80)
0.9% SS	3,490,271.70 (1,100,656.24) ^c^	-	5694.70 (1755.56) ^c^	18.90 (5.80)

Global comparison using the ANOVA test with Welch correction (*p* < 0.001) followed by the Games–Howell test. Read vertically, the same superscript letters show non-statistically significant differences. SIM: Simvastatin; DC: Diclofenac: NaOCl: sodium hypochlorite; SS: saline solution.

## Data Availability

Data will be available on request from the authors.
